# Identification of Parkinson’s disease-associated chromatin regulators

**DOI:** 10.1038/s41598-023-30236-4

**Published:** 2023-02-22

**Authors:** Hailong Xing, Shanshan Wang, Ke Li

**Affiliations:** 1 Department of Neurosurgery, Binzhou Medical University Hospital, Binzhou, Shandong China; 2 Department of Neurosurgery, Binzhou Central Hospital, Binzhou, Shandong China; 3 Department of Interventional Neurosurgery, Binzhou Medical University Hospital, Binzhou, 256603 Shandong China

**Keywords:** Computational biology and bioinformatics, Neuroscience

## Abstract

Parkinson’s disease (PD) is a common neurological disorder that causes quiescent tremors, motor delays, depression, and sleep disturbances. Existing treatments can only improve symptoms, not stop progression or cure the disease, but effective treatments can significantly improve patients’ quality of life. There is growing evidence that chromatin regulatory proteins (CRs) are involved in a variety of biological processes, including inflammation, apoptosis, autophagy, and proliferation. But the relationship of chromatin regulators in Parkinson’s disease has not been studied. Therefore, we aim to investigate the role of CRs in the pathogenesis of Parkinson’s disease. We collected 870 chromatin regulatory factors from previous studies and downloaded data on patients with PD from the GEO database. 64 differentially expressed genes were screened, the interaction network was constructed and the key genes with the top 20 scores were calculated. Then we discussed its correlation with the immune function of PD. Finally, we screened potential drugs and miRNAs. Five genes related to the immune function of PD, BANF1, PCGF5, WDR5, RYBP and BRD2, were obtained by using the absolute value of correlation greater than 0.4. And the disease prediction model showed good predictive efficiency. We also screened 10 related drugs and 12 related miRNAs, which provided a reference for the treatment of PD. BANF1, PCGF5, WDR5, RYBP and BRD2 are related to the immune process of Parkinson’s disease and can predict the occurrence of Parkinson’s disease, which is expected to become a new target for the diagnosis and treatment of Parkinson’s disease.

## Introduction

PD is a common neurodegenerative disease. The cause of PD is still unclear, and the progress of the disease cannot be stopped with existing medical methods. Although there is currently no cure for PD, effective treatment can significantly improve patients' quality of life^1,2^. With the rapid development of high-throughput sequencing data, many diseases have entered the stage of molecular diagnosis and treatment, and it also brings the possibility of curing some intractable diseases. Many biomarkers associated with Parkinson’s disease have been discovered: SNCA was the first gene found to be associated with Parkinson’s disease, and SCNA mutations increase the risk of sporadic Parkinson’s disease^3,4^. PRKN is a gene associated with autosomal recessive Parkinson’s disease^5,6^. LRRK2 is a related gene that causes autosomal dominant PD^7,8^. In addition, many biomarkers have been discovered and used in the treatment of Parkinson's disease, which plays an important role in the improvement of the diagnosis and treatment of Parkinson’s disease^9–12^. However, studies have also shown that the effects of many gene mutations may be related to race, which makes certain genes not universally applicable to the diagnosis and treatment of Parkinson's disease^13–15^. Therefore, it is urgent to search for more biomarkers.

CRs are a class of enzymes with specialized functional domains capable of recognizing, forming and maintaining epigenetic states in a cellular context-dependent manner. CRs are indispensable upstream regulators of epigenetics. According to their regulatory roles in epigenetics, CRs are generally classified into three categories: DNA methylation, histone modification, and chromatin remodeling factors. Aberrant expression of CRs is associated with various biological processes such as inflammation, apoptosis, autophagy, and proliferation, suggesting that dysregulation of CRs may lead to the development of various diseases. Therefore, CRs are expected to become new targets for the treatment of various diseases. At present, there is little research on the relationship between CRs and PD. In order to explore the relationship between the two and provide new ideas for the treatment of PD. Using biological analysis, we obtained CRs associated with PD immunity, and predicted related drugs and miRNAs.

## Results

### Identification of differentially expressed CRs

**Figure 1:**
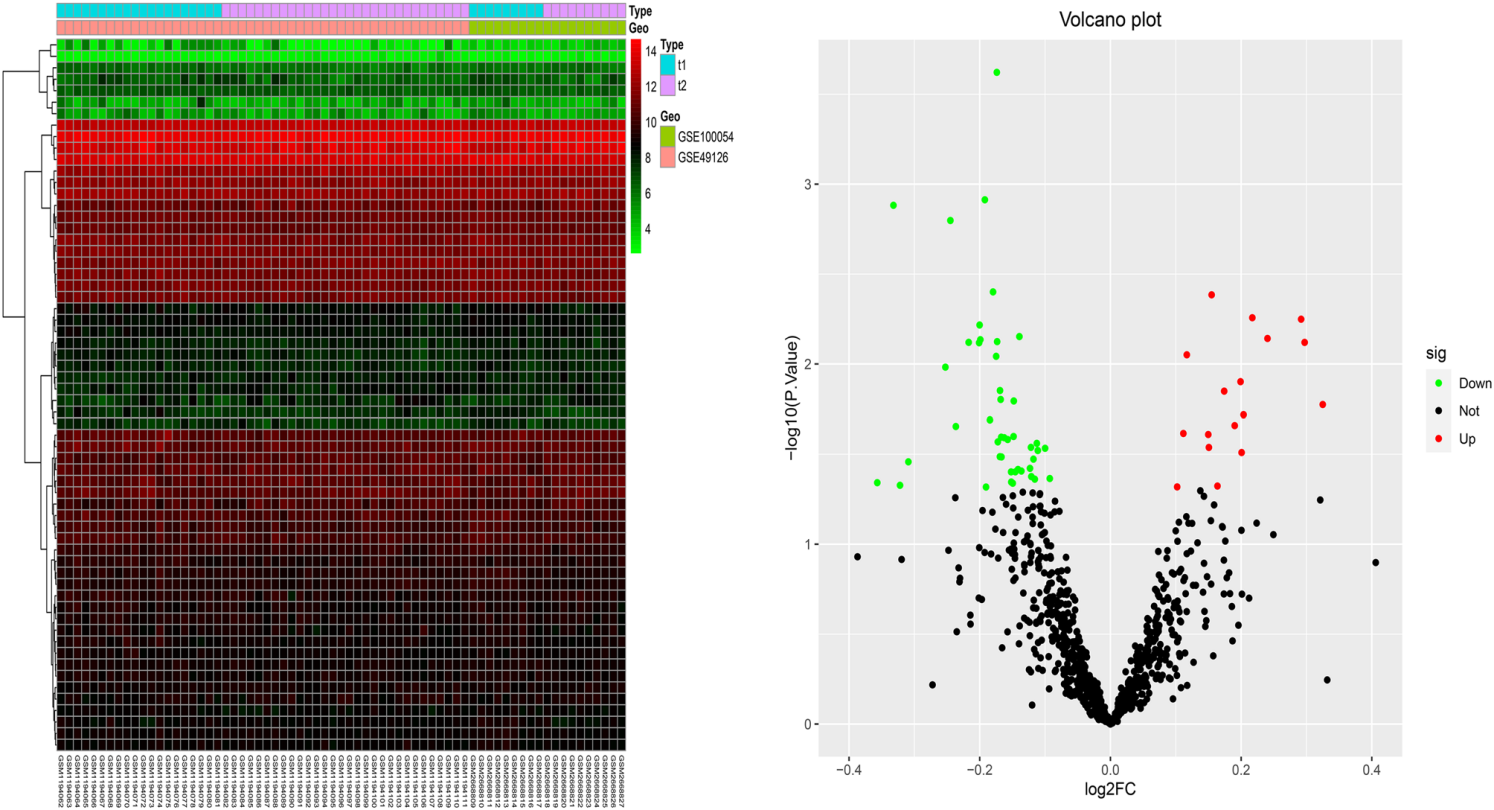
Heatmap and volcanic map showed differentially expressed CRs.

We screened 64 differentially expressed CRS genes from 29 normal samples and 40 PD patients. The differential CRs included 22 up-regulated genes and 42 down-regulated genes (Fig. 1).

### Enrichment analysis and protein–protein interaction (PPI) analysis of differential CRs

**Figure 2:**
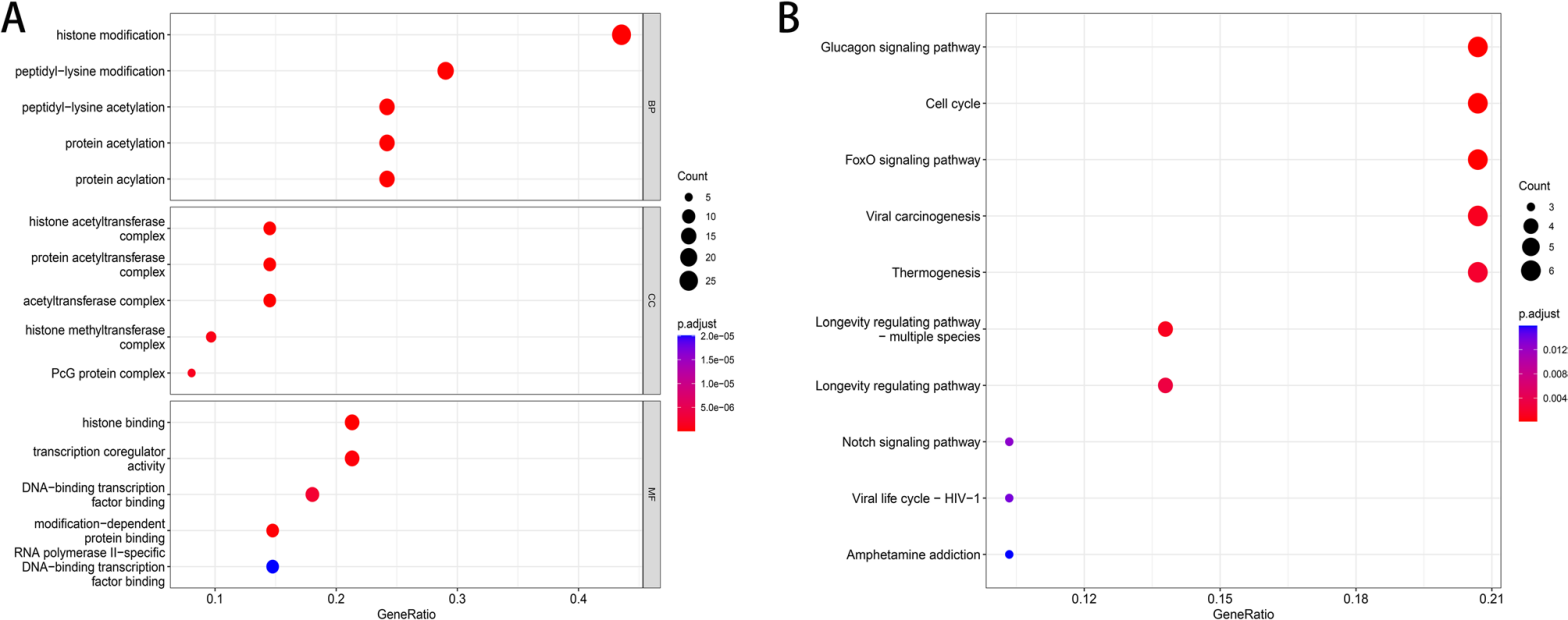
Enrichment analyses of differentially expressed CRs. (**A**) GO analysis; (**B**) KEGG analysis.

**Figure 3:**
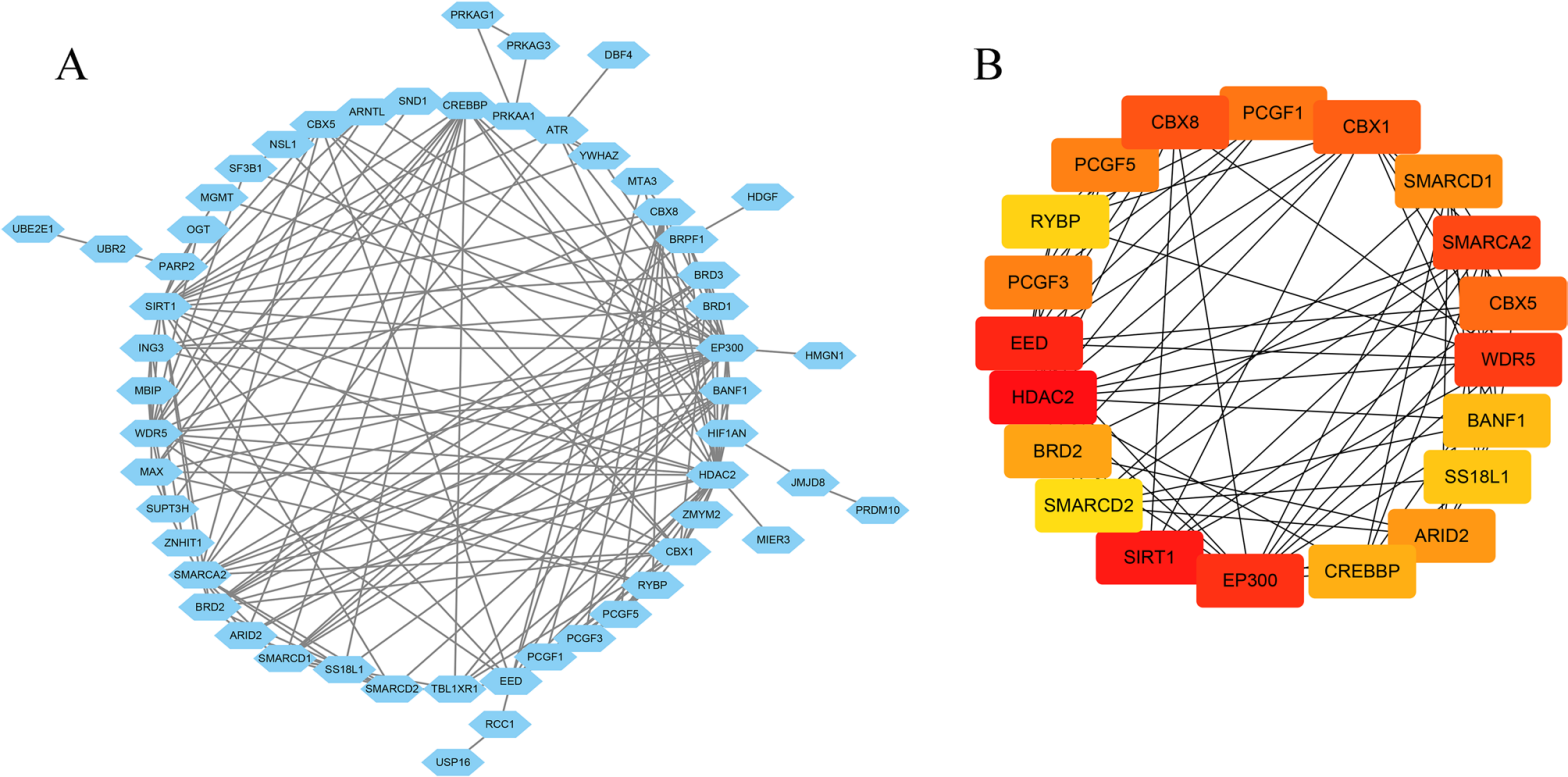
(**A**) Protein–protein interaction network of differentially expressed CRs; (**B**) The Hub genes.

For 64 differential genes, we carried out GO analysis and KEGG analysis. We could see the enrichment of genes in the corresponding biological processes and related pathways. In the GO analysis, we could see that CRs is mainly related to biological processes, such as histone modification, peptidyl-lysine modification, peptidyl–lysine acetylation, protein acetylation and protein acylation (Fig. 2A). In KEGG analysis, we found that differential CRs is mainly involved in FoxO signaling pathway, Glucagon signaling pathway, Viral carcinogenesis, Cell cycle and Thermogenesis (Fig. 2B). We analyzed the differential genes by PPI (Fig. 3A), and screened the Top 20 Hub genes by using the cytoHubba plug-in in Cytoscape software (Fig. 3B).

### Analysis of immune cells and immune function

**Figure 4:**
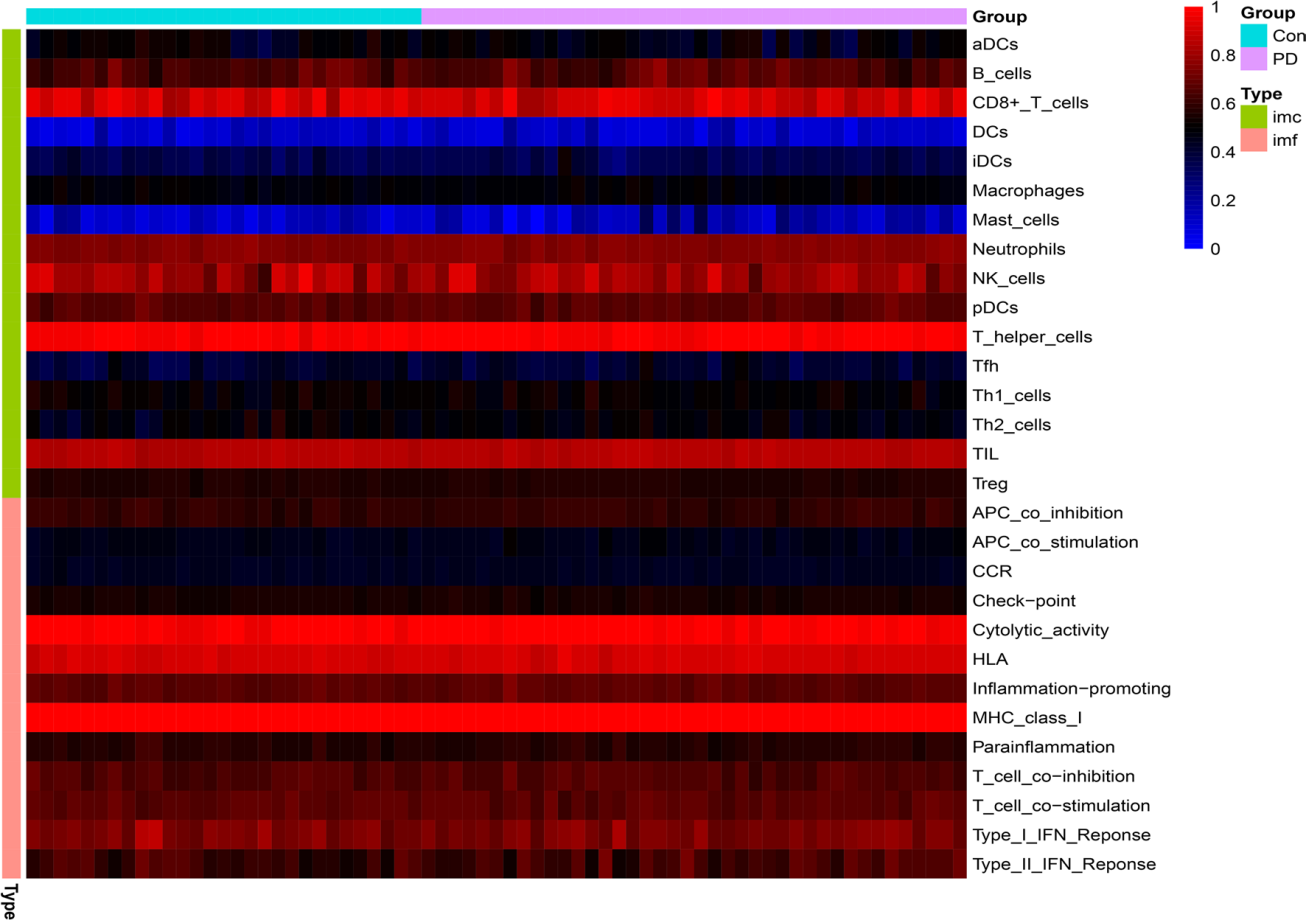
Heatmap showed the expression of immune cells and immune functions.

**Figure 5:**
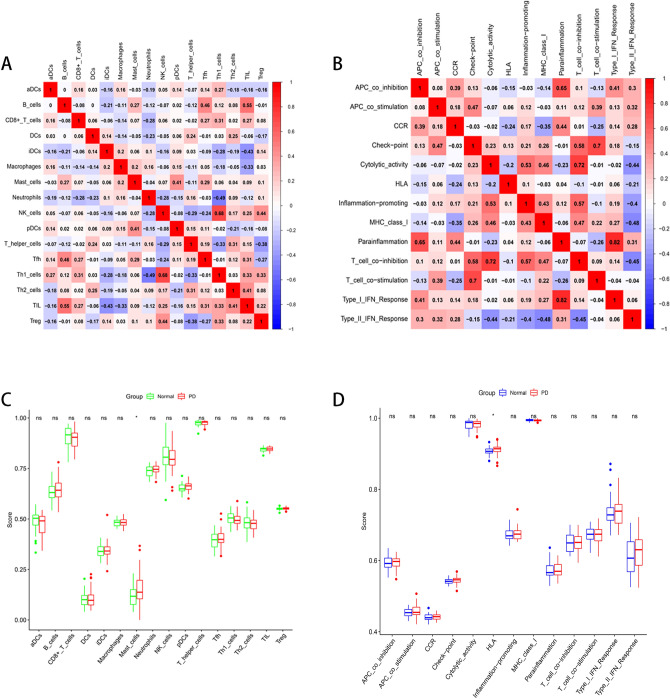
Analysis of immune cells and immune function. (**A**–**B**) Correlation analysis of immune cells and immune function; (**C**–**D**) Differences in immune cells and immune function between PD samples and normal samples.

We used ssGSEA algorithm to get the expression of related immune cells and immune function in the sample, and then we analyzed the correlation and difference of immune cells and immune function respectively (Fig. 4). We could see that in immune cells, the correlation between Th1 cells and Neutrophils is 0.68 and the correlation between TIL and B cells was 0.55, suggesting that they had strong positive correlation; while the correlation between Th1 cells and Mast cells is − 0.49 and the correlation between iDCs and TIL was − 0.45, suggesting that they had a strong negative correlation (Fig. 5A). In the correlation analysis of immune function, there was a strong positive correlation between Para inflammation and Type I IFN Response, between T cell co-inhibition and Cytolytic activity, and between Check-point and T cell co-stimulation, reaching 0.83, 0.72, 0.70 respectively. And there was a strong negative correlation between MHC class I and Type II IFN Response, between T cell co-inhibition and Type II IFN Response, and between Type II IFN Response and Cytolytic activity, reaching − 0.48, − 0.45, − 0.44 respectively (Fig. 5B). And we could see that there were significant differences in the immune cells Mast cells and immune function HLA between normal samples and PD patients (*P* < 0.05) (Fig. 5C–D).

### Construction of a nomogram

**Figure 6:**
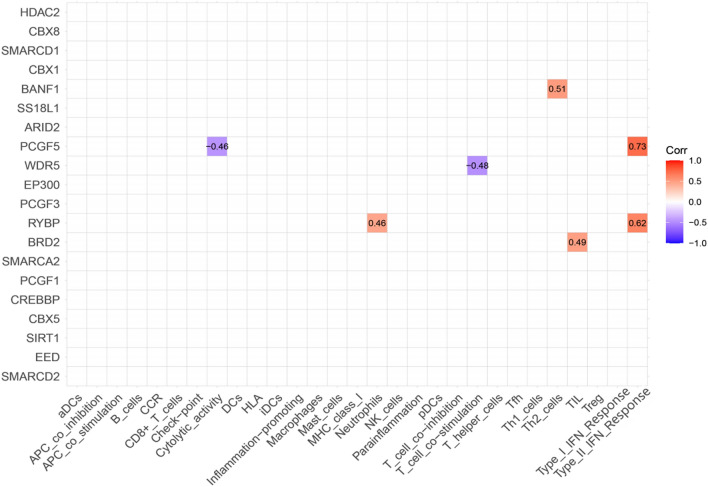
Correlation analysis of Hub genes with immune cells and immune function.

**Figure 7:**
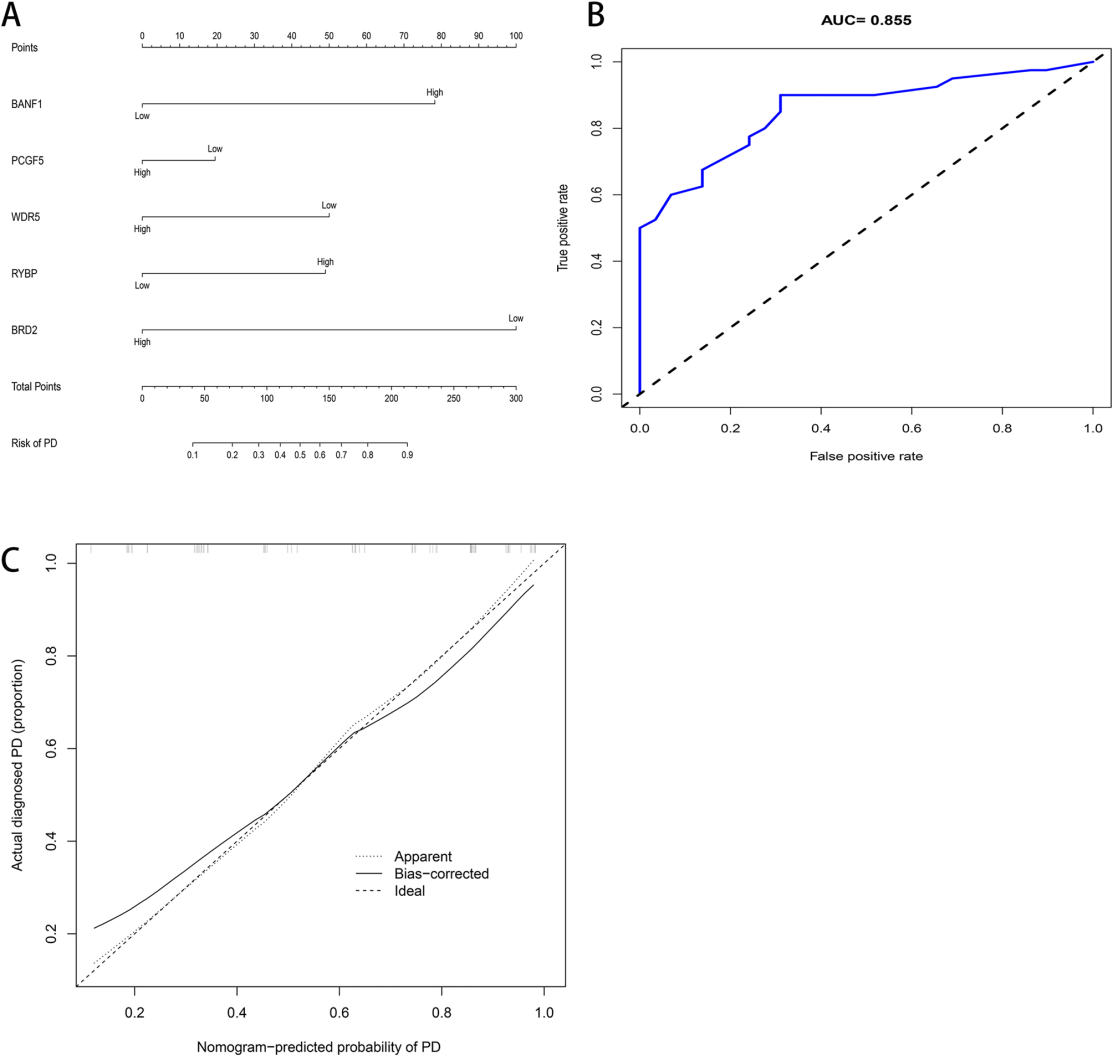
Construction of a nomogram. (**A**) Nomogram for predicting the risk of PD; (**B**) The ROC curve of prediction model; (**C**) The calibration plots for predicting the risk of PD.

We analyzed the correlation between 20 hub genes and immune cells and immune function, and finally identified 5 genes according to the absolute value of correlation greater than 0.4 (Fig. 6). Among them, BANF1 was positively correlated with Th2 cells, PCGF5 was positively correlated with Type II IFN Response, negatively correlated with Cytolytic activity, WDR5 was negatively correlated with T cell co-stimulation, RYBP was positively correlated with Neutrophils and Type II IFN Response. BRD2 was positively correlated with TIL. Then we constructed a predictive model to predict the possibility of various genes and PD (Fig. 7A–C). The nomogram shows that the higher the expression of BANF1 and RYBP, the higher the probability of developing PD, and the higher the expression of PCGF5, WDR5 and BRD2, the lower the probability of developing PD. And the Calibration curve and the Receiver Operating Characteristic (ROC) curve show that the model has good predictive function.

### Prediction of related drugs and miRNAs

**Figure 8:**
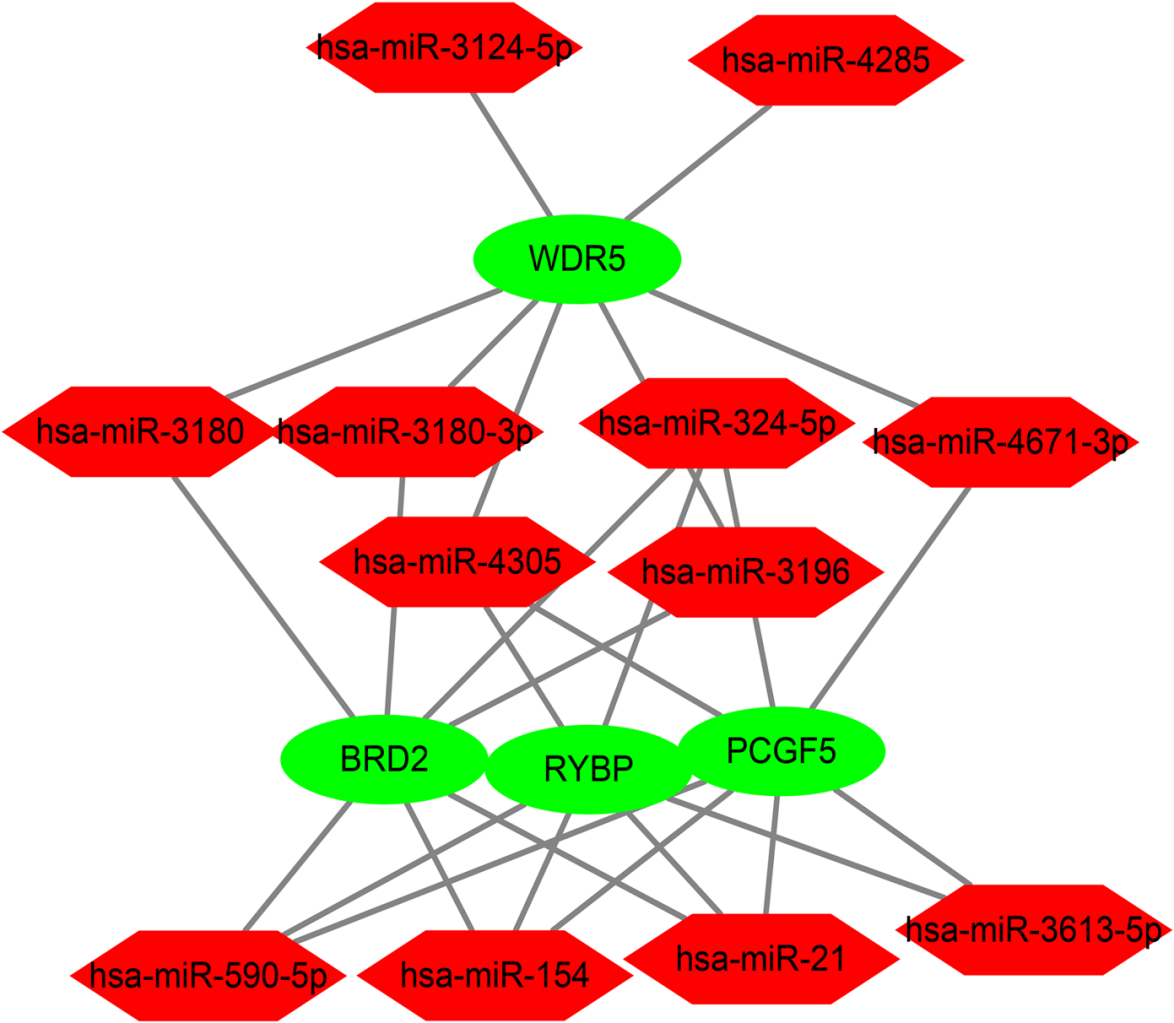
The network diagram of related miRNAs.

We uploaded immune-related hub genes to ENRICHR database, using DSigDB database to predict related drugs, and using TargetScan database to predict related miRNAs. We got 112 related drugs (The top ten are shown in Table 1) and 12 related miRNAs (Table 2), and use Cytoscape software to draw the network diagram (Fig. 8).

**Table 1:** The top 10 drugs of DSigDB database analyses results.

Term	*P* value	Genes
piperlongumine HL60 UP	8.27E−04	BRD2; RYBP
fulvestrant MCF7 DOWN	9.47E−04	BRD2; RYBP
parthenolide PC3 UP	0.001004724	BRD2; RYBP
nitrofural PC3 DOWN	0.001298126	BRD2; BANF1
chlortetracycline HL60 DOWN	0.001332121	BRD2; RYBP; BANF1
glibenclamide HL60 DOWN	0.002667555	BRD2; RYBP; BANF1
triazolam CTD 00006926	0.002996653	BRD2
midazolam CTD 00006334	0.003246051	BRD2
ouabain HL60 UP	0.003448524	BRD2; RYBP
VITAMIN E CTD 00006994	0.003824751	BRD2; RYBP; BANF1

**Table 2:** The 12 miRNAs of TargetScan database analyses results.

Term	*P* value	Genes
hsa-miR-4305	0.004286817	RYBP; PCGF5; WDR5
hsa-miR-154	0.006764147	BRD2; RYBP; PCGF5
hsa-miR-21	0.007389343	BRD2; RYBP; PCGF5
hsa-miR-590-5p	0.007389343	BRD2; RYBP; PCGF5
hsa-miR-324-5p	0.008096529	BRD2; RYBP; PCGF5
hsa-miR-3124-5p	0.032570286	WDR5
hsa-miR-3180	0.046782106	BRD2; WDR5
hsa-miR-3180-3p	0.046782106	BRD2; WDR5
hsa-miR-3196	0.046782106	BRD2; WDR5
hsa-miR-4285	0.047572617	WDR5
hsa-miR-3613-5p	0.049389696	RYBP; PCGF5
hsa-miR-4671-3p	0.049930191	PCGF5; WDR5

## Discussion

PD is a common neurodegenerative disease. Complex pathogenic factors and uncontrollable conditions make the treatment of PD difficult^16,17^. With the continuous development of medical technology, the treatment of PD has made great progress. Drug therapy is a common method for the treatment of PD. The drugs represented by levodopa are widely used in the treatment of PD^18,19^. Deep brain electrode stimulation, as a mainstream surgical method, also has a good curative effect^20,21^. Exercise rehabilitation therapy can be regarded as a convenient and low-cost method^22^. Recently, more and more evidence show that immune response is also involved in the pathogenesis of PD, so the immunotherapy of PD has a broad development prospect^23,24^. CRs are a recent class of enzymes with special functional domains. Previous studies have shown that chromatin regulatory factors are involved in a variety of cellular biological processes^25,26^, but there are few studies on the relationship between chromatin regulatory factors and PD.

In our study, we used bioinformatics analysis to screen 64 differentially expressed chromatin regulatory factors, and then we analyzed the differential CRs by GO analysis and KEGG analysis. In GO analysis, we can see that CRs is mainly related to biological processes, such as histone modification, peptidyl-lysine modification, peptidyl–lysine acetylation, protein acetylation and protein acylation. Most of these are epigenetic processes, and numerous studies have shown that epigenetic processes play an important role in neurodegenerative diseases such as Parkinson’s disease^27–30^. This also suggests a potential link between CRs and Parkinson’s disease. We analyzed the differential CRs by PPI, obtained the relationship between them, and screened 20 hub genes. Then we analyzed the relevant immune cells and immune function, we obtained the expression of immune cells and immune function in each sample, and carried out a series of analysis. According to the analysis, Th1 cells and Neutrophils, TIL and B cells are positively correlated, while Th1 cells and Mast cells, iDCs and TIL contents are negatively correlated. In the immune function, Para inflammation and Type I IFN Response, T cell co-inhibition and Cytolytic activity, Check-point and T cell co-stimulation had strong positive correlation; MHC class I and Type II IFN Response, T cell co-inhibition and Type II IFN Response, Type II IFN Response and Cytolytic activity had strong negative correlation. The content of Mast cells and HLA was different between pd samples and normal samples. Some studies have shown that histamine can regulate microglia and peripheral circulating monocytes in the brain to induce innate immune response and participate in the pathogenesis of Parkinson’s disease^31^. Other studies have shown that the activation of IFN- γ may be a potential link between Parkinson's disease and inflammation and neurodegeneration^32,33^. The expression of neuronal MHC-I also plays an important role in neurodegenerative diseases including Parkinson’s disease^34^. CGAS/STING-IFN-I signal mediates neuroinflammation in the pathological process of Parkinson’s disease^35^. Most of the immune functions we have analyzed have been confirmed to be closely related to Parkinson's disease, but the related immune functions may not be confirmed, which also needs further research to explore its specific mechanism. In order to further analyze the relationship between CRs and immunity, we carried out correlation analysis on hub gene and immunity, and finally obtained five immune-related CRs, including BANF1, PCGF5, WDR5, RYBP and BRD2. BANF1 is a nuclear envelope protein involved in a variety of biological processes such as mitosis, viral infection, chromatin and gene regulation, and DNA damage responses. It plays an important role in many cancers such as stomach cancer and breast cancer^36–38^. PCGF5 is a polycomb protein, which plays an important role in regulating the differentiation of embryonic stem cells into neural progenitor cells^39^, some studies have also shown that it is related to T-cell leukemia^40^. WDR5 is involved in the regulation of gene expression, which is not only related to a variety of cancers such as prostate cancer^41^, breast cancer^42^, liver cancer^43^, but also related to Huntington's chorea^44^, rheumatoid arthritis^45^ and other diseases. Brd2 is a Histone Acetyl Transferase (HAT), which plays an important role in the treatment of PD and other neurodegenerative diseases by inducing histone H3K27 acetylation and leading to chromatin opening and enhancing neuronal gene expression^46,47^. RYBP is a member of the PcG protein family, which can bind to Ring1 protein, ubiquitin itself and play the role of E3 ubiquitin ligase, and then mediate gene silencing. RYBP plays an important role in regulating gene expression and cell function. It has been proved that there is a certain correlation between RYBP and many kinds of tumors^48–50^. Except for BRD2, there are few studies on the relationship between the other four genes and PD. Our study suggests that these five CRs may be associated with PD, which provides some ideas for the immunotherapy of PD.

Of course, our research also has some shortcomings. First of all, we have obtained five CRs related to Parkinson's disease through analysis, but not at the protein level. Animal experiments are also one of the limiting factors. Secondly, the specific mechanism of the related genes in Parkinson’s disease needs to be verified by further experiments.

## Conclusions

In conclusion, we obtained the CRs that may play a role in the immunity of PD through bioinformatics analysis, which provides some ideas for the treatment of PD, and its specific mechanism needs to be verified by experiments.

## Methods

### Data download and identification of differentially expressed CRs

We downloaded the PD gene expression dataset GSE1000541^51^ and GSE491262^52^ (Gene Expression, Omnibus, GEO, https://www.ncbi.nlm.nih.gov/geo/) from the public database GEO, including 29 normal patient samples and 40 PD patients’ samples. The gene expression profiles of the two data sets were normalized by eliminating the batch effect. We also retrieved 870 CRs (CRS)^53^ from previous studies. According to the criteria of |logFC| > 0.1 and *P* Value < 0.05. We used R software to screen differentially expressed CRs. We drew gene expression heat maps and volcano maps to visualize the expression of differential genes between different samples.

### Functional enrichment analysis and protein–protein interaction (PPI) analysis of differential CRs

We performed Gene Ontology (GO) analysis and Kyoto Encyclopedia of Genes and Genomes (KEGG) analysis^54–56^ of differentially expressed CRs. The standard of significant enrichment is adj. *P* value < 0.05. We submit the differentially expressed CRs to the STRING database (http://www.string-db.org/) to obtain detailed information about gene interaction. We import the file into Cytoscape software^57^ to screen out TOP20 gene as Hub gene by cytoHubba plug-in.

### Using single sample gene set enrichment analysis (ssGSEA) algorithm to obtain the expression of related immune cells and immune function in the sample and analyze the correlation

In order to understand the differences in the expression of immune cells and immune function between patients with PD and normal subjects, we calculated the abundance of immune cells and immune function in related samples by using ssGSEA algorithm, and drew the relevant heatmap. We analyzed the correlation between immune cells and immune function, and revealed the correlation between different immune cells and different immune functions, and then we analyzed the differences between immune cells and immune functions. To explore whether there are differences in different immune cells and immune functions between normal samples and patients with PD.

### Screening Hub genes related to immunity and constructing predictive model

Through the analysis of the screened hub gene and immune cells and immune function, we screened out the immune-related hub gene. Then we used hub genes related to immunity to build a prediction model, build a nomogram, predicted the probability of related genes and disease occurrence, and used the calibration curve to evaluate the prediction accuracy of nomogram.

### Prediction of drugs and miRNAs related to immune-related hub gene

We screened the immune-related hub genes, uploaded them to the ENRICHR database (https://maayanlab.cloud/Enrichr/), and predicted the related drugs and miRNAs through the related database.

## Data Availability

All data generated or analyzed during this study are included in this published article.

## Abbreviations

PD: Parkinson’s disease
CRs: Chromatin regulators
PPI: Protein–protein interaction
GO: Gene ontology
KEGG: Kyoto encyclopedia of genes and genomes
ssGSEA: Single sample gene set enrichment analysis
ROC: Receiver operating characteristic
HAT: Histone acetyl transferase
